# Comparison of in-hospital outcomes and long-term survival for valve-in-valve transcatheter aortic valve replacement versus the benchmark native valve transcatheter aortic valve replacement procedure

**DOI:** 10.3389/fcvm.2023.1113012

**Published:** 2023-02-09

**Authors:** Anthony Matta, Laszlo Levai, Jerome Roncalli, Meyer Elbaz, Frederic Bouisset, Vanessa Nader, Stephanie Blanco, Francisco Campelo Parada, Didier Carrié, Thibault Lhermusier

**Affiliations:** ^1^Department of Cardiology, Toulouse University Hospital, Toulouse, France; ^2^Department of Cardiology, Hôpitaux Civils de Colmar, Colmar, France

**Keywords:** TAVR, valve in valve implantation, redo aortic operation, degenerated bioprosthesis, transcatheter and surgical aortic valve replacement

## Abstract

**Background:**

In recent years, the number of patients with failed surgically implanted aortic bioprostheses and the number of candidates for valve-in-valve transcatheter aortic valve replacement (VIV-TAVR) have been increasing.

**Objectives:**

The purpose of this study is to evaluate the efficacy, safety, and long-term survival outcomes of VIV-TAVR compared with the benchmark native valve transcatheter aortic valve replacement (NV-TAVR).

**Methods:**

A cohort study was conducted on patients who underwent TAVR in the department of cardiology at Toulouse University Hospital, Rangueil, France between January 2016 and January 2020. The study population was divided into two groups: NV-TAVR (*N* = 1589) and VIV-TAVR (*N* = 69). Baseline characteristics, procedural data, in-hospital outcomes, and long-term survival outcomes were observed.

**Results:**

In comparison with NV-TAVR, there are no differences in TAVR success rate (98.6 vs. 98.8%, *p* = 1), per-TAVR complications (*p* = 0.473), and length of hospital stay (7.5 ± 50.7 vs. 4.4 ± 2.8, *p* = 0.612). The prevalence of in-hospital adverse outcomes did not differ among study groups, including acute heart failure (1.4 vs. 1.1%), acute kidney injury (2.6, 1.4%), stroke (0 vs. 1.8%, *p* = 0.630), vascular complications (*p* = 0.307), bleeding events (0.617), and death (1.4 vs. 2.6%). VIV-TAVR was associated with a higher residual aortic gradient [OR = 1.139, 95%CI (1.097–1.182), *p* = 0.001] and a lower requirement for permanent pacemaker implantation [OR = 0.235 95%CI (0.056–0.990), *p* = 0.048]. Over a mean follow-up period of 3.44 ± 1.67 years, no significant difference in survival outcomes has been observed (*p* = 0.074).

**Conclusion:**

VIV-TAVR shares the safety and efficacy profile of NV-TAVR. It also represents a better early outcome but a higher non-significant long-term mortality rate.

## Introduction

The world’s population is aging and, in parallel, prolonged life expectancy after cardiovascular surgery with the limited durability of bioprosthetic valves has actively contributed to a continuous increase in the prevalence of symptomatic severe aortic stenosis and the number of patients with failed bioprosthetic aortic valves (1–3). Accordingly, the number of candidates for cardiac reinterventions is expected to increase in the near future. Transcatheter aortic valve replacement (TAVR) was initially introduced as a less invasive approach for high-surgical-risk aortic stenosis patients. It was then proposed as a safe alternative therapeutic option for those who are at intermediate and even low risk of needing cardiac surgery (4–6). A few years ago, TAVR was also revealed as an important treatment for bioprosthetic structural valve degeneration. To date, there have been no randomized clinical trials that compare the outcomes and survival outcomes of redo-surgical aortic valve replacement versus TAVR in patients with failed bioprosthetic valves. Available data in literature are limited to observational retrospective cohorts, registries, and meta analysis. An advantage of valve-in-valve TAVR (VIV-TAVR) over repeat surgical aortic valve replacement in early survival outcomes and post-survival complications has been reported in patients with degenerated bioprosthetic valves (7–9). However, higher rates of myocardial infarction, hospital readmissions, and patient-prosthesis mismatch have been observed after VIV-TAVR (7–9). Based on the latest guidelines, VIV-TAVR is considered a complex intervention associated with the high risk of per-procedural and post-procedural complications and should be performed by an experienced heart team following a multidisciplinary decision (10, 11). In the present study, we compare the in-hospital outcomes and long-term survival outcomes of VIV-TAVR patients versus native valve TAVR (NV-TAVR) patients.

## Materials and methods

### Study population and design

A cohort study was conducted on 1,658 consecutive patients who underwent TAVR at Toulouse University Hospital between January 2016 and January 2020. The indication of TAVR and the choice of valve type (self-expandable vs. balloon-expandable) were based on the Heart Team’s clinical judgment, in the best interests of the patient at the time of the procedure. The study population was divided into two groups: VIV-TAVR vs. NV-TAVR. We evaluated the rate of TAVR success; per-procedural complications, like aortic rupture, cardiac tamponade, device migration or embolization, immediate post-TAVR paravalvular leak, and post-TAVR residual aortic gradient; and in-hospital post-TAVR adverse outcomes, including vascular complications, bleeding, acute kidney injury, pacemaker implantation, stroke, acute heart failure, and death. TAVR success was defined by the correct device position and the absence of surgical conversion and/or per-procedural death. Vascular complications, bleeding, and acute kidney injury were defined according to the Valve Academic Research Consortium (VARC-2) criteria (12). Post-TAVR paravalvular leak and trans-aortic gradient were evaluated by transthoracic echocardiography before hospital discharge. We also compared the length of hospital stay and prevalence of prolonged CCU stay (>24 h) between study groups. Lastly, we assessed the living status (alive or dead) of each of the study’s participants by 1 September 2022.

### Data collection and endpoint

Data concerning baseline characteristics of the study population, characteristics of TAVR procedures (vascular access, type of implanted valve, contrast volume, procedure duration, per-procedural adverse outcomes, vascular access closure device), in-hospital post-TAVR complications (as previously defined), in-hospital mortality, transthoracic echocardiography parameters, and length of hospital stay were collected from the Orbis and Hemolia database systems of Toulouse University Hospital. The living status of study participants was observed by September 2022. The aim of this study is to assess the TAVR success rate, the prevalence of in-hospital post-TAVR complications, and the long-term survival outcomes of VIV-TAVR compared with NV-TAVR.

### Statistical analysis

Categorical variables were represented by number and percentage and compared using the Chi-square test or Fisher exact test, as appropriate. The continuous variables were represented by mean and standard deviation and compared using the *t*-student test. The normality and homoscedasticity tests for quantitative variables were performed. A multivariable logistic regression was performed to evaluate the association of in-hospital post-TAVR complications with VIV-TAVR compared to NV-TAVR. The Kaplan-Meier curve and the Log-rank test were performed for long-term survival analysis. A two-sided *p*-value less than 0.05 was considered of statistical significance. Statistical analyses were conducted using SPSS version 20 (IBM Corp., Armonk, NY, USA).

## Results

**FIGURE 1:**
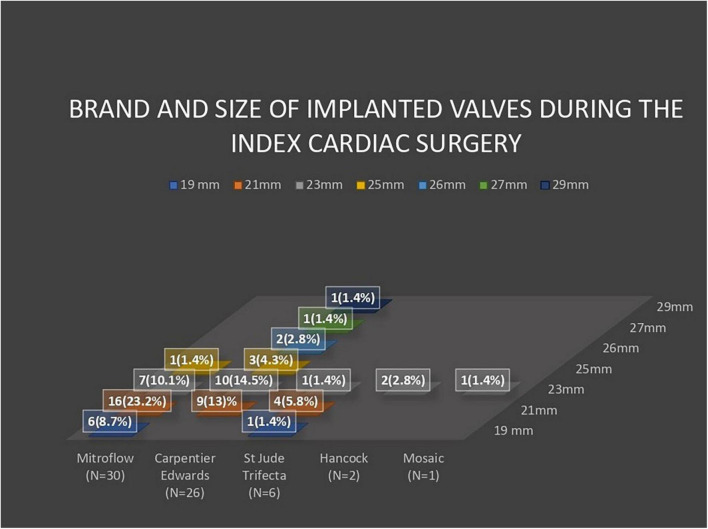
Histogram showing the brand and size of implanted valves during index cardiac surgery.

**FIGURE 2:**
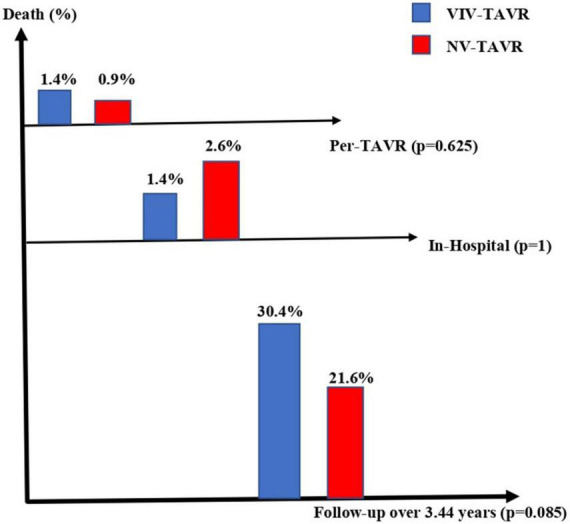
Kaplan-Meier survival outcome analysis in patients with VIV-TAVR versus NV-TAVR.

**FIGURE 3:**
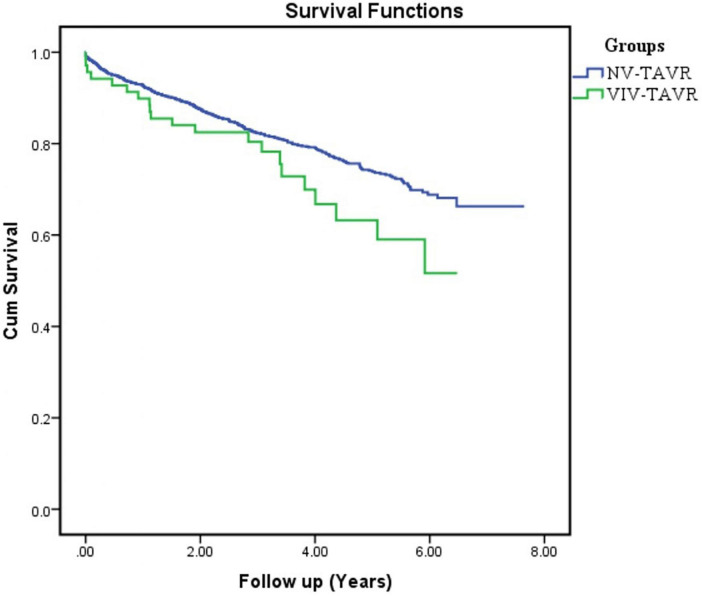
Timeline of the progression of all-cause mortality rate in patients with VIV-TAVR versus NV-TAVR.

Of the 1,658 patients included in this study, 69 (4.2%) study participants underwent TAVR for failed surgically implanted bioprosthetic aortic valves (VIV-TAVR group), while 1,589 (95.8%) study participants underwent TAVR for severe aortic stenosis with native aortic valves (NV-TAVR group). The brands of valves implanted during the index cardiac surgery were Mitroflow (*N* = 30, 43.5%), Carpentier Edwards (*N* = 26, 37.7%), St Jude Trifecta (*N* = 6, 8.7%), Hancock (*N* = 2, 2.8%), and Mosaic (*N* = 1, 1.4%) (Figure 1). The size and brand of degenerated bioprosthesis were unidentified in four study participants (5.8%). Characteristics of the study population are shown in Table 1. The mean age of the study population was 83.9 ± 7.1-years and 50.2% of participants were women. Pre-TAVR coronary angiography revealed significant coronary artery disease defined by a more than 50% reduction in coronary lumen in 33.3% of the study population, and 23.2% were subsequently treated with PCI during pre-TAVR work-up. There were no significant differences between the VIV-TAVR and NV-TAVR groups accounting for gender, comorbidities, and cardiovascular risk factors. However, study participants in the VIV-TAVR group were younger (80.4 ± 12.2 vs. 84.1 ± 6.8), with more history of cardiovascular events, and expressed a higher predicted mortality risk. The success rate of the VIV-TAVR procedure was as high as NV-TAVR (98.6 vs. 98.8%). Most TAVR procedures were performed through the common femoral artery (92.8%), and Proglide was mainly used as a vascular access closure device (89%) (Table 2). Unlike the NV-TAVR group, where a balloon-expandable valve was commonly used (54.8%), the majority of implanted devices in the VIV-TAVR group were self-expandable (95.7%). The mean duration of VIV-TAVR interventions was longer than that of NV-TAVR procedures (87 ± 24 vs. 76 ± 24 min, *p* < 0.05), but VIV-TAVR was performed with a low mean contrast volume (114 ± 49 vs. 138 ± 49 ml, *p* < 0.05). The mean of residual aortic gradient tends toward a significantly higher level in association with VIV-TAVR (12.3 ± 6.7 vs. 10.6 ± 5.2, *p* = 0.053). Otherwise, the prevalence of per-TAVR complications, post-TAVR echocardiographic paravalvular leak, and length of hospital and CCU stays did not differ between the two study groups. Focusing on in-hospital post-TAVR complications, the single significant difference was less permanent pacemaker implantation in patients with degenerated bioprosthetic valves (2.9 vs. 11.7%, *p* = 0.024) (Table 3). Note that stroke did not occur in the VIV-TAVR group (0 vs. 1.8%). The multivariable logistic regression confirmed the negative association of pacemaker implantation with VIV-TAVR [OR = 0.235 95%CI (0.056–0.990), *p* = 0.048] and revealed the positive association of residual aortic gradient with VIV-TAVR [OR = 1.139, 95%CI (1.097–1.182), *p* = 0.001] (Table 4). Thus, no significant differences in vascular complication, bleeding, acute heart failure, stroke, and acute kidney injury were observed. In contrast to the predictions, the in-hospital mortality rate was lower in the VIV-TAVR group than in the NV-TAVR group (1.4 vs. 2.6%) but did not reach a statistically significant level (Table 3). Over a mean follow-up period of 3.44 ± 1.67 years, the observed rate of all-cause death was 22%, and it was non-significantly higher in the VIV-TAVR group than in the NV-TAVR group (30.4 vs. 21.6%, *p* = 0.085) (Figure 2). Lastly, the Kaplan-Meier curve and the Log-rank test showed no difference in survival outcome analysis over time (*p* = 0.074) (Figure 3).

**TABLE 1:** Baseline characteristics of study population.

	Study population (*N* = 1658)	VIV-TAVR (*N* = 69)	NV-TAVR (*N* = 1589)	*p*-value
Age (years)	83.9 ± 7.1	80.4 ± 12.2	84.1 ± 6.8	0.015
BMI (kg/m^2^)	26.3 ± 5.4	25.6 ± 5	26.3 ± 5.4	0.264
Women	833 (50.2%)	35 (50.7%)	798 (50.2%)	0.935
Dyslipidaemia	752 (45.4%)	37 (53.6%)	715 (45%)	0.159
Diabetes mellitus	454 (27.4%)	17 (24.6%)	437 (27.5%)	0.601
Arterial hypertension	1161 (70%)	44 (63.8%)	1117 (70.3%)	0.247
Smoker	49 (3%)	3 (4.3%)	46 (2.9%)	0.455
Chronic lung disease	319 (19.2%)	10 (14.5%)	309 (19.4%)	0.307
Atrial fibrillation	641 (38.7%)	24 (34.8%)	617 (38.8%)	0.499
Prior stroke	127 (7.7%)	13 (18.8%)	114 (7.2%)	0.001
Prior myocardial infarction	131 (7.9%)	6 (8.7%)	125 (7.9%)	0.803
Prior CABG	103 (6.2%)	10 (14.5%)	93 (5.9%)	0.004
PCI during pre-TAVR work-up	384 (23.2%)	17 (24.6%)	367 (23.1%)	0.766
Logistic Euro SCORE	15 ± 11.9	23.9 ± 13.8	14.6 ± 11.7	0.05
STS-PROM	5.8 ± 5.1	7.3 ± 4.9	5.8 ± 5.1	0.02

VIV-TAVR, valve-in-valve transcatheter aortic valve replacement; NV-TAVR, native valve transcatheter aortic valve replacement; BMI, body mass index; PCI, percutaneous coronary intervention; Euro SCORE, European system for cardiac operative risk evaluation; STS-PROM, society of thoracic surgeons predicted risk of mortality.

**TABLE 2:** Characteristics of transcatheter aortic valve replacement (TAVR) procedures.

	Study population (*N* = 1658)	VIV-TAVR (*N* = 69)	NV-TAVR (*N* = 1589)	*p*-value
Transfemoral access	1539 (92.8%)	67 (97.1%)	1472 (92.6%)	0.230
Valve type				<0.001
Self-expandable	783 (47.3%)	66 (95.7%)	717 (45.2%)	
Balloon-expandable	874 (52.7%)	3 (4.3%)	872 (54.8%)	
Proglide as vascular access closure device	1475 (89%)	63 (91.3%)	1412 (88.9%)	0.694
Contrast volume (ml)	137 ± 49	114 ± 49	138 ± 49	<0.001
Procedure duration (min)	76 ± 23	87 ± 24	76 ± 24	<0.001
TAVR success rate	1639 (98.8%)	68 (98.6%)	1571 (98.8%)	1
Per-TAVR complications				0.473
Death	15 (0.9%)	1 (1.4%)	14 (0.9%)	
Device migration/embolization	12 (0.7%)	0 (0%)	12 (0.8%)	
Aortic annular rupture	7 (0.4%)	0 (0%)	7 (0.4%)	
Cardiac tamponade	17 (1%)	0 (0%)	17 (1.1%)	
Post-TAVR PVL	104 (6.3%)	2 (2.9%)	102 (6.4%)	0.315
Prolonged CCU stay (›24 h)	307 (18.5%)	11 (15.9%)	296 (18.6%)	0.574
Length of hospital stay (days)	7.4 ± 49.5	4.4 ± 2.8	7.5 ± 50.7	0.612

VIV-TAVR, valve-in-valve transcatheter aortic valve replacement; NV-TAVR, native valve transcatheter aortic valve replacement; PVL, paravalvular leak; CCU, cardiac care unit.

**TABLE 3:** In-hospital post-TAVR complications and mortality rate.

	Study population (*N* = 1658)	VIV-TAVR (*N* = 69)	NV-TAVR (*N* = 1589)	*p*-value
Acute heart failure	18 (1.1%)	1 (1.4%)	17 (1.1%)	0.537
Acute kidney injury	42 (2.5%)	1 (1.4%)	41 (2.6%)	1
Stroke	29 (1.7%)	0 (0%)	29 (1.8%)	0.630
Vascular complications				0.307
Minor	111 (6.7%)	7 (10.1%)	104 (6.5%)	
Major	71 (4.3%)	4 (5.8%)	67 (4.2%)	
Bleeding events				0.617
Minor	43 (2.8%)	3 (4.3%)	43 (2.7%)	
Major	42 (2.5%)	2 (2.9%)	40 (2.5%)	
Pacemaker implantation	188 (11.3%)	2 (2.9%)	186 (11.7%)	0.024
In-hospital death	42 (2.5%)	1 (1.4%)	41 (2.6%)	1

VIV-TAVR, valve-in-valve transcatheter aortic valve replacement; NV-TAVR, native valve transcatheter aortic valve replacement.

**TABLE 4:** Adjusted multivariate logistic regression testing the association between VIV-TAVR and adverse outcomes.

	OR	95%CI	*p*-value
Pacemaker implantation	0.235	(0.056–0.990)	0.048
Mean residual aortic gradient	1.139	(1.097–1.182)	0.001
Acute heart failure	4.317	(0.483–38.596)	0.191
Stroke			0.998
Major vascular complication	1.298	(0.333–5.060)	0.707
Major bleeding event	0.584	(0.062–5.509)	0.639

## Discussion

The main finding of the present study is that VIV-TAVR interventions result in a similar success rate to NV-TAVR, as well as fewer permanent pacemaker implantations and a higher residual aortic gradient. Furthermore, VIV-TAVR patients are less likely to be exposed to different post-TAVR complications, at least during the hospital stay, accounting for vascular complications, bleeding events, and stroke. Far from the predicted mortality, VIV-TAVR showed a better early mortality rate and no significant difference in long-term, real-world survival.

In our study, the self-expandable device was nearly implanted in almost all VIV-TAVR patients. A recently published study evaluating the outcomes of self-expandable valves versus balloon-expandable valves in both VIV-TAVR and NV-TAVR patients has reported better results from the self-expandable device implant for degenerated aortic bioprostheses (13). The overall findings in our study are in line with previously reported data from valve-in-valve registries (14, 15) and published cohort studies (13, 16). For example, the prevalence of new permanent pacemaker implantation in the present study was closer to that reported in a large cohort including 1,150 patients with failed surgical aortic valve replacement in the VIV-TAVR (2.9 vs. 3%) and NV-TAVR (11.7 vs. 10.9%) groups, respectively (16). In comparison with NV-TAVR, the TAVR device is placed within the bioprosthetic valve during VIV-TAVR intervention. Thereby, the limited contact with the myocardium results in less stress on the cardiac conduction system and is likely to explain why fewer patients are requiring permanent pacemaker implantation following the VIV-TAVR procedure (17). It is of note that pacemaker implantation after TAVR is associated with higher risks of all-cause mortality and rehospitalization for heart failure but is not associated with stroke or endocarditis (18). However, a high residual gradient following the VIV-TAVR procedure remains a real concern. Pre-existing prosthesis-patient mismatch, small effective orifice area, and deep valve implant were identified as strong predictors, while supra-annular valve type and high transcatheter heart valve implant were associated with risk reduction (19). In addition, a decrease in mean residual gradient and an improvement in mean aortic valve area were associated with bioprosthetic valve fracture in the context of VIV-TAVR for patients with degenerated bioprosthetic valves (20). The present study shows a significantly higher mean residual aortic gradient after VIV-TAVR procedures. However, the ratio of residual aortic gradient within the study groups was lower than that previously reported (16), largely due to a larger number of self-expandable valve implants being used in patients with failed surgical bioprostheses. A higher residual gradient after VIV-TAVR is highly likely due to a small effective orifice area after the procedure, and it is highly dependent on the valve implanted previously during the index cardiac surgery. We emphasize that the success of VIV-TAVR depends on the size of the valve implanted during the index cardiac surgery and, sometimes, surgeons end up implanting very small valves that increase the risk of mismatch. This could be solved by using techniques to enlarge the aortic annulus/root to implant larger valves during index cardiac surgery (21, 22). Note, the benefit of aortic annulus enlargement on reducing moderate to severe patient-prosthesis mismatch after surgical aortic valve replacement should be assessed against a potential increase in perioperative mortality (21). Avoiding mismatch during index cardiac surgery is of paramount importance to reducing mortality after surgical aortic valve replacement and to the success of future VIV-TAVR (21–24). Furthermore, the size and brand of the surgically implanted bioprosthesis, in conjunction with the final result of the index cardiac surgery, are relatively important in determining the outcomes of VIV-TAVR. Lastly, VIV-TAVR procedures showed a similar safety and efficacy profile to NV-TAVR. In terms of survival, it was associated with better early outcomes but a higher long-term all-cause mortality rate. In parallel, an immediate protective effect was also revealed while comparing VIV-TAVR to redo-surgery as a therapeutic option for the management of degenerated aortic bioprostheses (9, 25). However, a recent meta analysis noticed the reverse of this observed early advantage of VIV-TAVR on all-cause death in favor of redo surgery after 6 months of follow-up (26), whereas a propensity-matched cohort study reported a better 5-year survival outcome after VIV-TAVR versus redo surgery (25).

### Limitation

The study design may be predisposed to selection bias. The choice between redo surgery and VIV-TAVR in patients presenting with failed surgically implanted bioprostheses depends on the local heart team’s decision that was deemed the best option for the patient at the time of procedure. The limited number of balloon-expandable valve implants in the VIV-TAVR group makes it difficult to compare the outcomes of the two types of valves and to guide the choice of device in patients with degenerated bioprostheses. We reported all-cause mortality because we are not able to identify the cause of death in all study participants, and subsequently, we are also unable to report the cardiovascular mortality rate. The study population is relatively elderly, with a mean age of 83.9 years. This finding may influence the observed outcomes considering the potential role of frailty, malnutrition, and sarcopenia (27). However, almost all study participants benefited from geriatric assessment before undergoing the procedure. We also know that VIV-TAVR and NV-TAVR are not interchangeable procedures. Therefore, our purpose was to evaluate the efficacy and safety of VIV-TAVR compared to the benchmark NV-TAVR procedure, which has proven its effectiveness in randomized clinical trials, and not to find out which one is superior. We recognize the inherent differences in populations, and we sought to verify whether VIV-TAVR is comparable in safety and efficacy to NV-TAVR. Therefore, comparing VIV-TAVR with redo-SAVR would be the definitive test for comparative effectiveness.

## Conclusion

In the absence of randomized clinical trials comparing VIV-TAVR to redo surgical aortic valve replacement, this study provides insights into the safety and efficacy of VIV-TAVR procedures, compared to the standard NV-TAVR, and the treatment modality of choice in high-risk severe aortic stenosis patients. The present findings suggest that high-risk patients with failed bioprosthetic valves do reasonably well after a VIV-TAVR procedure in a real-world setting.

## Data availability statement

The raw data supporting the conclusions of this article will be made available by the authors, without undue reservation.

## Author contributions

All authors listed have made a substantial, direct, and intellectual contribution to the work, and approved it for publication.
